# The ecometric properties of a measurement instrument for prospective risk analysis in hospital departments

**DOI:** 10.1186/1472-6963-14-103

**Published:** 2014-03-03

**Authors:** Steffie M van Schoten, Rebecca J Baines, Peter Spreeuwenberg, Martine C de Bruijne, Peter P Groenewegen, Jop Groeneweg, Cordula Wagner

**Affiliations:** 1 NIVEL – Netherlands Institute for Health Services Research, Otterstraat 118-124, PO Box 1568, Utrecht 3500 BN, Netherlands; 2 Department of Public and Occupational Health & EMGO Institute for Health and Care Research, Vrije Universiteit Medical Center (VUmc), Amsterdam, The Netherlands; 3 Department of Sociology, Department of Human Geography, Utrecht University, Utrecht, Netherlands; 4 Faculty of Social and Behavioural Sciences, Cognitive Psychology, Leiden University, Leiden, Netherlands; 5 TNO, Hoofddorp, Netherlands

**Keywords:** Prospective risk, Prospective risk analysis, Risk factors, Ecometric, Patient safety, Adverse event, Hospitals

## Abstract

**Background:**

Safety management systems have been set up in healthcare institutions to reduce the number of adverse events. Safety management systems use a combination of activities, such as identifying and assessing safety risks in the organizational processes through retrospective and prospective risk assessments. A complementary method to already existing prospective risk analysis methods is Tripod, which measures latent risk factors in organizations through staff questionnaires. The purpose of this study is to investigate whether Tripod can be used as a method for prospective risk analysis in hospitals and whether it can assess differences in risk factors between hospital departments.

**Methods:**

Tripod measures risk factors in five organizational domains: (1) Procedures, (2) Training, (3) Communication, (4) Incompatible Goals and (5) Organization. Each domain is covered by 15 items in the questionnaire. A total of thirteen departments from two hospitals participated in this study. All healthcare staff working in the participating departments were approached. The multilevel method ecometrics was used to evaluate the validity and reliability of Tripod. Ecometrics was needed to ensure that the differences between departments were attributable to differences in risk at the departmental level and not to differences between individual perceptions of the healthcare staff.

**Results:**

A total of 626 healthcare staff completed the questionnaire, resulting in a response rate of 61.7%. Reliability coefficients were calculated for the individual level and department level. At the individual level, reliability coefficients ranged from 0.78 to 0.87, at the departmental level they ranged from 0.55 to 0.73. Intraclass correlations at the departmental level ranged from 3.7% to 8.5%, which indicate sufficient clustering of answers within departments. At both levels the domains from the questionnaire were positively interrelated and all significant.

**Conclusions:**

The results of this study show that Tripod can be used as a method for prospective risk analysis in hospitals. Results of the questionnaire provide information about latent risk factors in hospital departments. However, this study also shows that there are indications that the method is not sensitive enough to detect *differences* between hospital departments. Therefore, it is important to be careful when interpreting differences in potential risks between departments when using Tripod.

## Background

Patient safety is an important aspect of the quality of care in hospitals. Patient safety can be defined as the reduction of the risk of unnecessary harm associated with healthcare to an acceptable minimum [1]. Previous studies have shown that between 2.9% and 16.6% of hospital admissions lead to adverse events [2], of which approximately 50% are potentially preventable [3]. Adverse events can be defined as harm to patients that was not caused by the underlying disease but by medical management, leading to prolonged hospitalization, re-hospitalization, disability or death [1]. In order to reduce the number of adverse events, safety management systems have been set up in healthcare institutions [4-6]. Safety management systems aim to prevent undesired outcomes in healthcare by a combination of activities, such as improvement projects, incident reporting and analyses, and risk assessments to identify and assess safety risks in the organizational processes [6].

Risk assessments can be performed retrospectively or prospectively. To date, retrospective risk management has been most common in healthcare [7]. Several methods for retrospective risk assessments in healthcare are currently used; the key element in all these methods is the analysis of the *causes* of incidents, near-misses and unsafe situations in order to prevent them from happening again in the future. Examples of these methods are the Prevention and Recovery Information System for Monitoring and Analysis (PRISMA) and Root Cause Analysis (RCA) [7,8]. These methods have two disadvantages. First, they require an open incident reporting culture since they rely on reporting by healthcare staff. Second, the analysis can only take place *after* an unsafe situation has been revealed, with or without consequences [7]. It therefore makes sense to analyze risks in a prospective manner, complementary to retrospective methods, to prevent unsafe environments that could potentially lead to adverse events [7].

In prospective risk analysis, processes are analyzed for potential risks in order to prevent errors from happening in the first place. Some well-known methods are the Healthcare Failure Mode (HFMEA) and Bow-Tie [9-13]. Both methods focus on analysis of care processes, and are often organized around a specific disease and therefore bound to one particular medical specialty. In these methods, a group of professionals meets several times to systematically map out the care process that was chosen for the risk analysis. The potential risks and their consequences are determined, and ways of preventing these situations are considered. Although these methods can create awareness about potential risks, they are time-consuming and only focus on one care process at a time.

A complementary method to existing prospective risk analysis methods is Tripod, which takes account of the key organizational processes. Tripod measures latent risk factors categorized into what are termed Basic Risk Factors (BRFs) at the departmental level by means of staff questionnaires. Latent risk factors are risk factors that are present within departments but are not always clearly visible. Tripod uses the individual risk perception of staff to determine the BRFs for an individual department. BRFs are used to determine potential risks in five general organizational domains: Procedures, Training, Communication, Incompatible Goals, and Organization. The method has its origins in the petrochemical sector [14-16] but could also be applied in the healthcare setting [17]. Tripod has the potential to be broader and less time-consuming than existing methods. The method can be used as a starting point to obtain a broad picture of the level of control over the organizational processes at the departmental level, and the results allow prioritization of further in-depth prospective risk analyses. The purpose of this study is to investigate whether Tripod is appropriate as a method for prospective risk analysis within hospital departments. This study is based on the adjusted Tripod for prospective risk analysis in healthcare, known as Tripod Delta HC. We will discuss the changes to Tripod to create Tripod Delta HC in more detail in the Methods section. The research questions addressed in this study are:

•*Can Tripod Delta HC be used as a measurement instrument for prospective risk analysis in hospitals?*

•*Can Tripod Delta HC be used to assess differences in latent risk factors between hospital departments?*

## Methods

### Measurement instrument: theory and development

Tripod is founded on the idea that human error can be prevented or mitigated by controlling the environment people work in [14,16,18-20]. According to Tripod, there are latent failures in every work environment and these can be categorized into BRFs. Each of the BRFs may contribute to adverse events in different ways, and when a combination of different undesirable situations emerge at the same time, this will lead to disturbances in the operational process, with or without consequences [21,22]. BRFs are controllable in the sense that they can be influenced by changing the organization and management of processes.

Tripod was originally developed for use in the petrochemical industry but is also seen as a promising option for patient safety [5,20]. However, the original questionnaire could not be applied in healthcare without some modifications to create ownership of the users in a healthcare setting [23]. In this study, we took the shortened Tripod questionnaire, known as Tripod Delta Lite, as a starting point for the development of Tripod Delta HC. This shortened questionnaire, which contained five general BRFs and consisted of 75 items, was extracted from the full version that contained ten further specific risk factors and a total of 150 items.

Modifications to Tripod Delta Lite were made by the authors, who consulted a group of 14 experts. The group consisted of registered nurses and researchers with experience in healthcare who assessed the content validity of the questionnaire by reviewing the modified questionnaire. Experts were asked to evaluate the importance and content of each item in the questionnaire and state whether they thought any important aspects were missing. Comments and suggestions were summarized. Nurses generally suggested changes to improve the applicability of the item in a healthcare setting. Researchers commented on the structure of the questionnaire and response categories. This led to the use of five-point scales rather than dichotomous (yes/no) response categories that were used in Tripod Delta Lite. The modifications did not alter the underlying assumptions of the items or the underlying constructs of the questionnaire and led to a revised questionnaire that was used in this study and is called Tripod Delta HC. In the remainder of this article we will only refer to this modified healthcare version.

### Measurement instrument: structure

Tripod Delta HC measures BRFs in five organizational domains: (1) Procedures, (2) Training, (3) Communication, (4) Incompatible Goals and (5) Organization. Each BRF is covered by 15 items that form a scale. Table 1 gives the definitions of the different BRFs and some example items.

**Table 1:** Definitions of the five basic risk factors of Tripod Delta HC

***Basic Risk Factors***	***Definition***	***Example item***
BRF Procedures	Insufficient quality or availability of procedures, guidelines, instructions, and manuals (specifications, administration, use in practice).	“Because procedures are insufficiently clear, I sometimes have to act according to my own discretion”.
BRF Training	No or insufficient competence or experience among healthcare staff (not sufficiently suited to their tasks, inadequately trained).	“There are always sufficiently experienced healthcare staff present in the department”.
BRF Communication	No or ineffective communication between the various sites, departments or healthcare staff of an organization or with the official bodies.	“Important information is often sent to the wrong department in the hospital”.
BRF Incompatible Goals	The situation in which healthcare staff must choose between optimal working methods according to the established rules on one hand, and the pursuit of production, financial, political, social or individual goals on the other.	“Necessary maintenance work has been postponed due to high costs”.
BRF Organization	Shortcomings in the organization’s structure, organization’s philosophy, organizational processes or management strategies, resulting in inadequate or ineffective management of the organization.	“The tasks are not properly coordinated between departments so that work is carried out twice”.

(Controlling the controllable, Jop Groeneweg 2002).

In total the questionnaire contains 75 items that are measured on a five- point Likert scale ranging from ‘totally disagree’ to ‘totally agree’. Respondents were also offered the response option of ‘not applicable’: this box could be checked if the participant was unfamiliar with the content of the item or had no opinion. Respondents were asked to answer the questions with the last six months in mind. In addition, some background characteristics were measured (i.e. age, gender, years working in hospital). See Additional file 1 for the items used.

### Sampling

This study was carried out at one academic hospital and one general hospital, based on a convenience sample. Three departments in the academic hospital took part and ten departments in the general hospital, making thirteen departments in total. There was a wide variety of departments. All healthcare staff working in the participating departments at the time of the study were approached, regardless of whether they had daily patient interaction. The Dutch Medical Research Involving Human Subjects Act does not apply to this research. Therefore, no approval was needed from the Medical Ethics Committee.

### Procedure

Data was collected between December 2011 and March 2012. Each hospital appointed one contact, who was the quality coordinator in both cases. The contact invited all the hospital’s departments to participate in our study. In both hospitals, *all* departments were approached and asked to participate. However, in the academic hospital, the participation of departments was voluntary and the decision of a department to participate lay with the head of that department. In the general hospital, the director made the decision that all departments needed to participate in the study. The e-mail addresses of all healthcare staff currently working in the participating departments were collected. To guarantee privacy, the researcher did not receive the personalized e-mail addresses. The two hospitals followed different procedures to ensure this. In the academic hospital, invitation e-mails were sent to the secretaries of the departments, who forwarded invitation e-mails to the individual e-mail addresses. In the general hospital, the e-mail addresses were encrypted by personnel number. The invitation e-mail contained the purpose of the study and information about the procedure and guaranteed anonymity of the respondent. As participation was voluntary, no written informed consent needed to be obtained from the participants. The e-mail contained a link that led to the questionnaire simply by clicking on it. Reminder e-mails were sent to non-respondents after two and four weeks. The questionnaire took approximately 15–20 minutes to complete. In total, 1015 persons were invited and 626 healthcare staff completed the questionnaire, resulting in a response rate of 61.7%. Table 2 gives an overview of the response rates per hospital. Table 3 gives an overview of characteristics of the study population and shows that the largest group of respondents consists of registered nurses (47.4%).

**Table 2:** Response rates

	***Invited***	***Completed***	***Response***
	***(n)***	***(n)***	***(%)***
Hospital A- academic	332	195	58.7
Hospital B- general	683	431	63.1
Total	1015	626	61.7

**Table 3:** Overview of the study population

		***N***	***%***
Gender	Male	120	19.2
	Female	505	80.8
Age	<30	110	17.7
	30-50	350	56.2
	>50	163	26.2
Department	Children’s department	48	7.7
	Emergency room	33	5.3
	Gynecology	69	11.0
	Intensive care department	48	7.7
	Internal department	29	4.6
	Lung diseases & cardio department	35	5.6
	Neurology	92	14.7
	Operating rooms	57	9.1
	Orthopedic department	16	2.6
	Short-stay nursing department	42	6.7
	Surgical department	26	4.2
	Thorax center	131	20.9
Experience	0-5 years	230	36.7
	6-20 years	266	42.1
	>20 years	130	20.8
Profession	Nurse	297	47.4
	Physician	43	6.9
	Other (interns, operation assistants etc.)	286	45.7
Patient contact	Yes	549	87.7
	No	77	12.3

Note: Neurology was included in both hospitals.

### Data preparation

First, negatively worded items were recoded so that a lower score reflected a lower potential risk for all items. In general, respondents were more likely to agree that a positive situation applied than a negative situation. Data was checked for completeness. All items in the questionnaire were mandatory and respondents were not able to skip questions, although they could exit the questionnaire before the end. A total of 32 respondents (5.1% of the total sample) did not answer at least 50% of the questions, meaning that they had stopped partway through the questionnaire; these respondents were excluded from further analyses. The remaining dataset did not contain any missing data. When respondents checked the category ‘not applicable', the score was replaced by a missing value. The analyses were performed on the remaining 594 respondents.

### Ecometrics approach

Ecometrics is a multilevel method to evaluate the validity and reliability of imperfect measures of contextual properties [24]. An ecometrics approach was used to ensure that the differences between departments are attributable to differences in potential risks at the departmental level and not to differences between the individual perceptions of the healthcare staff who responded to the questionnaire. The aim of this method is to measure the latent characteristics of ecological units (in this research, the ecological unit is the hospital department). Furthermore, the method aims to combine multiple observations into one scale to analyze the reliability and validity of the scale. The data structure is as follows: the items are at the lowest level, nested within the healthcare staff member, and healthcare staff are nested within the departments, which are at the highest level. There were 13 departments in our sample, which were treated as separate units in the analyses. A weighted item average for all healthcare staff was calculated for each item to calculate an average scale value. This was done by using the item weights for the fixed effects. The item variance, which is an indication of the measurement error, was taken into account in this analysis.

### Statistical procedure

Aspects of the reliability of the scales were assessed in terms of internal consistency using a reliability coefficient. Reliability indicates how well the individual items of a scale measure the underlying concept of the scale. The interpretation is the same at the individual level as at the departmental level, and comparable to Cronbach’s alpha coefficient [24,25]: values range between 0 and 1, with a higher score representing a more reliable scale. If the reliability coefficient for a scale is at least 0.70, this suggests that the items in a scale are measuring the same concept. Values above 0.80 indicate high internal consistency. Descriptive analyses were used to calculate means, standard deviations and the range of scores for the different scales in the questionnaire at the individual and departmental levels. Variance and intraclass correlations were calculated to assess the clustering of answers at the individual and departmental levels [24]. An intraclass correlation of 20% is seen as moderate [26]. Correlations between scales were calculated at the individual and departmental levels to check that the scales were measuring different concepts. High correlations between scales indicate that the scales are measuring similar concepts. The minimum required sample size to assure adequate reliability for comparing results between departments was estimated for each of the scales, based on the number of healthcare staff in each department and reliability coefficients in the current study. This gives the minimum number of respondents per BRF that is needed to make reliable inferences about differences between departments. The descriptive analyses were conducted using STATA version 11.0 and the multilevel analyses were performed using MlwiN version 2.24.

## Results

### Reliability analyses

Table 4 gives the reliability coefficients for the different scales. At the individual level, the reliability coefficients ranged from 0.78 to 0.87. This indicates good to excellent internal consistency at the individual level. The internal consistency at the departmental level ranged from 0.55 to 0.73. For the BRF Procedures and the BRF Incompatible Goals the internal consistency of the scales at the departmental level was less than 0.70, which is below the minimum preferred value.

**Table 4:** Reliability of scales at the individual level and departmental level

***BRF***	***Reliability***	***Reliability***
	***Individual level***	***Departmental level***
BRF Procedures	0.87	0.68
BRF Training	0.83	0.70
BRF Communication	0.79	0.71
BRF Incompatible Goals	0.80	0.55
BRF Organization	0.78	0.73

Note: 0.70 or higher can be assumed sufficient.

### Descriptives of the scales

Table 5 gives the descriptive statistics for the scale scores at the individual and departmental levels, calculated using the multilevel model. On a scale from 1 to 5 (a lower score reflects a lower potential risk), the mean scale scores at the individual level ranged from 2.12 for the BRF Training to 2.89 for the BRF Procedures. At the departmental level, the mean scale scores ranged from 2.12 for the BRF Training to 2.92 for the BRF Procedures.

**Table 5:** Descriptive statistics of the basic risk factors at the individual and departmental levels on a scale from 1 to 5

***BRF***	***Individual level (N = 588)***		***Departmental level (N = 13)***	
	**Mean (SD)**	**Range**	**Mean (SD)**	**Range**
BRF Procedures	2.89 (0.47)	1.42 - 4.11	2.92 (0.11)	2.68 - 3.09
BRF Training	2.12 (0.41)	1.14 - 4.05	2.12 (0.10)	1.92 - 2.28
BRF Communication	2.49 (0.33)	1.51 - 4.01	2.49 (0.09)	2.29 - 2.59
BRF Incompatible Goals	2.16 (0.36)	0.99 - 3.42	2.17 (0.06)	2.01 - 2.24
BRF Organization	2.64 (0.36)	1.49 - 3.83	2.63 (0.11)	2.38 - 2.82

### Variance and intraclass correlations

The clustering of responses at the individual and departmental levels for each of the BRFs is shown in Table 6. The intraclass correlations (ICCs) at the departmental level ranged from 3.7% for the BRF Incompatible Goals to 8.5% for the BRF Organization. The ICC of 8.5% for the BRF Organization means that 8.5% of the variance in the responses to the various items in the BRF Organization can be attributed to differences between departments. In this study, clustering effects are relatively small. In the case of all the dimensions (BRFs) of Tripod Delta HC, most of the variance was at the individual level.

**Table 6:** Variance at the individual and departmental levels and intraclass correlation coefficients (ICCs)

	***Individual level***	***Departmental level***	
	**Variance**	**Variance**	**ICC%**
	**(SE)**	**(SE)**	
BRF Procedures	0.241***	0.016	6.36%
	(0.017)	(0.009)	
BRF Training	0.184***	0.014	6.85%
	(0.013)	(0.008)	
BRF Communication	0.121***	0.010*	7.57%
	(0.009)	(0.005)	
BRF Incompatible Goals	0.158***	0.006	3.70%
	(0.012)	(0.004)	
BRF Organization	0.150***	0.014*	8.53%
	(0.011)	(0.007)	

*p < 0.05. ** p < 0.01. ***p < .0.001 (significance was tested using the Wald statistic).

### Correlations between scales

The correlations between BRFs were examined in order to test the interdependency of the five different scales of Tripod Delta HC. The results are shown in Table 7. At both the individual and departmental levels, BRFs were positively correlated and all correlations were significant (P < 0.05). At the individual level, the correlations between BRFs ranged between 0.40 and 0.75. At the departmental level, the correlations ranged between 0.29 and 0.83. At both levels there was heterogeneity in the size of the correlations. Most correlations were stronger at the departmental level than the individual level. This indicates that the average responses at the departmental level are strongly correlated. For example, a department that scores highly for the BRF Organization is likely to have a high score for the BRF Communication as well.

**Table 7:** Correlations at the individual level (left of the diagonal) and departmental level (right of the diagonal)

	***BRF Procedures***	***BRF Training***	***BRF Communication***	***BRF Incompatible Goals***	***BRF Organization***
BRF Procedures	-	0.29	0.62	0.80	0.60
BRF Training	0.40	-	0.70	0.61	0.71
BRF Communication	0.56	0.53	-	0.81	0.83
BRF Incompatible Goals	0.64	0.59	0.62	-	0.81
BRF Organization	0.56	0.61	0.65	0.75	-

All correlations were significant at p < 0.05.

### Minimum required sample size for acceptable reliability at the departmental level

Table 8 gives the minimum required sample size for acceptable reliability at the departmental level. For each of the BRFs, the reliability coefficient is calculated for any given sample size. This makes it possible to determine how many healthcare staff per department need to be included in the study in order to make reliable interferences about differences between departments. For example, including 75 healthcare staff in a study results in a reliability coefficient of 0.82 for the BRF Procedures but the reliability for the BRF Incompatible Goals is 0.70 with 75 healthcare staff.

**Table 8:** Minimum required sample size for acceptable reliability at the departmental level*

***Number of respondents***	***BRF Procedures***	***BRF Training***	***BRF Communication***	***BRF Incompatible Goals***	***BRF Organization***
	**Reliability coefficient**				
20	0.54	0.55	0.57	0.38	0.59
25	0.60	0.60	0.62	0.43	0.64
30	0.64	0.65	0.66	0.48	0.69
40	0.70	0.71	0.72	0.55	0.74
50	0.75	0.75	0.76	0.60	0.78
75	0.82	0.82	0.83	0.70	0.84
100	0.86	0.86	0.87	0.75	0.88
125	0.88	0.88	0.89	0.79	0.90
150	0.90	0.90	0.91	0.82	0.92
200	0.92	0.92	0.93	0.86	0.94
250	0.94	0.94	0.94	0.88	0.95

*A reliability coefficient of less than 0.70 is considered below the minimum value for acceptable reliability.

## Discussion

This study assessed whether Tripod Delta HC can be used as a measurement instrument for prospective risk analysis in healthcare, and whether it can detect differences in risk factors between hospital departments. Most studies in healthcare use classical psychometric methods originating from psychology for assessing the reliability and validity of questionnaires, for example research into patient safety climate [27,28]. However, in healthcare these methods are only suitable for assessing differences in *individual* perceptions or attitudes. This study is mainly interested in the characteristics of the ecological construct (the department), and less in individual attitudes. Using individuals to assess the characteristics of departments results in imperfect measures because it is an indirect way to assess a department characteristic [24]. Therefore, this study used ecometrics to filter out this individual component. The individual variance is split from the variance at the departmental level and the remaining variance at the departmental level can be used as an indication for differences between departments. The results of this study show that the variance at the departmental level is small, although significant for some BRFs. This could have several causes. First, there might actually be only small differences between departments in the study population. Our study population consisted of only two hospitals; it could be that the departments within the hospitals were made very similar by organization-wide policies. In terms of patient safety, this is positive as it indicates that departments are similar and obtain low scores for the different BRFs. Second, there could be a large spread in the individual scores which would increase the variance at the individual level. However, we found no indication for this in our data. And lastly, Tripod Delta HC might not be sensitive enough to detect differences between departments. Given the small number of hospitals in this study, we are unable to determine the precise cause. However, this study does point out that it is important to take account of the variance at the higher level when measuring ecological units. Good psychometric properties are needed to describe differences in individual scores (healthcare staff), but when the intention of a measurement instrument is to describe differences in compositional scores (departments), good ecometric properties are needed.

### Practical implications

The results of this study show that the reliability of Tripod Delta HC is acceptable and that the questionnaire can be used to assess potential risks in hospitals. However, some of the BRFs exhibit lower reliability and little variance at the departmental level. For these BRFs it is important to be careful when drawing conclusions about differences between departments in potential risks. The minimum required sample size for acceptable reliability should be considered when using Tripod Delta HC in healthcare. This can be used as a tool to improve the reliability of the scales but also to prevent the unnecessary inclusion of healthcare staff in a study. As can be seen from Table 8, a larger number of respondents is needed for the BRF Incompatible Goals to assure good reliability than for the other BRFs. In practice, hospital departments are generally not large enough to deliver these numbers of healthcare staff. In these cases, the scores should not be used to compare departments with respect to this BRF. Furthermore, when developing interventions to improve patient safety, it is important to consider whether they need to be implemented at the individual level or the departmental level. As all BRFs show low variance at the departmental level, it would be more effective to implement interventions at the individual level throughout the hospitals.

### Limitations

We acknowledge several limitations to this study. First, only two hospitals were included in this study. The results cannot be generalized to the wider hospital population without caution. Future research, for instance in a larger group of hospitals, is needed to validate the findings of the current study. Second, the recruitment of departments was different in the two participating hospitals. However, the questionnaires were filled out by the healthcare staff and the decision to participate in both hospitals was individual and voluntary. Furthermore, the aim of this study was not to compare the two hospitals, but to validate the questionnaire that was used. Therefore, we are confident that the difference in numbers of participating departments between the hospitals did not affect the results of this study. Third, the largest group of respondents was registered nurses (47.4%; see Table 3). The group of physicians in our study was too small for separate statistical analyses. Fourth, questions in the questionnaire were grouped per BRF. This was meant to make it more convenient for the respondent to fill in the questionnaire, not having to switch mentally between concepts. It would be interesting to test whether a mix of questions from different concepts can improve the sensitivity of reliability measures. Although improving Tripod Delta HC was not the purpose of this study, future research could study the practical usage, for example by excluding items with low sensitivity or study the meaning of differences in correlations between departments with similar specialties from different hospitals.

## Conclusions

This research assessed whether Tripod Delta HC can be used as a method for prospective risk analysis in hospitals and whether it can measure differences between departments in their assessed risks, using a multilevel ecometric approach. Tripod Delta HC measures five BRFs at the hospital departmental level. In general, the ecometric properties of the modified healthcare version of Tripod Delta HC are satisfactory. The results show that the reliability of the instrument, as measured by the internal consistency of the items, is good at both the individual and departmental levels for most of the BRFs. This indicates that Tripod Delta HC can be used as a measurement instrument for prospective risk analysis in hospitals. However, two BRFs did not show much clustering at the departmental level or acceptable internal consistency at the departmental level. It was not possible to detect differences between departments for these BRFs. Overall, the results show that Tripod Delta HC is a useful instrument for assessing latent risks at the individual level, and for some BRFs at the departmental level as well. It might not be possible to measure differences between departments with Tripod Delta HC but further research in larger settings is needed to confirm these findings.

## Competing interests

The authors declare that they have no competing interests.

## Authors’ contributions

SS carried out the data collection, analyses and interpretation of the analyses, and drafted the manuscript. RB was involved in the design of the study and the pilot study, and helped to draft the manuscript. PS carried out the statistical analyses and reviewed the parts of the manuscript that involved the statistical analyses. MB was involved in the design of the study and critically revised the manuscript. PG was involved in the design of the study and critically revised the manuscript. JG was involved in the design of the study, advised on the analyses, and critically revised the manuscript. CW was involved in the design of the study and reviewed the manuscript. All authors were involved in revising and approving the final manuscript and accept responsibility for the data presented.

## Pre-publication history

The pre-publication history for this paper can be accessed here:

http://www.biomedcentral.com/1472-6963/14/103/prepub

## Supplementary Material

Additional file 1 TRIPOD Delta Health Care: questionnaire © NIVEL and Stichting Tripod Foundation. Click here for file
