# Supplementary material for: The ecometric properties of a measurement instrument for prospective risk analysis in hospital departments
Source: BMC Health Serv Res. 2014 Mar 3;14:103. doi: 10.1186/1472-6963-14-103 (PMC4233624; doi:10.1186/1472-6963-14-103)
Supplement: Additional file 1 — TRIPOD Delta Health Care: questionnaire © NIVEL and Stichting Tripod Foundation. [file 1472-6963-14-103-S1.docx]

**Additional File 1**

**TRIPOD Delta Health Care: questionnaire**

**Welcome to the risk assessment Tripod Delta HC**

In this questionnaire we will inquire about your experiences with regard to a number of possible risk factors in your department. The questionnaire contains 75 statements and can be answered in about 15 minutes. Before starting with filling in the questionnaire we would like to first request some background information from you.

| 1. **Background questions**   The questions below will assist us with the analysis and interpretation of the questionnaire. | | | |
| --- | --- | --- | --- |
| A1. | What is the year of your birth? |  | |
| A2. | What sex are you? | - female - male | |
| A3. | How long have you been working at this hohospital? | - less than 3 months - 3 months to 1 year - 1 to 5 years - 6 to 10 years - 11 to 15 years - 16 to 20 years - 21 years or more | |
| A4. | How long have you been working in your current department? | - less than 3 months - 3 months to 1 year - 1 to 5 years - 6 to 10 years - 11 to 15 years - 16 to 20 years - 21 years or more | |
| A5. | In which department in this hospital do you mainly work? | - Internal medicine - Cardiology - Neurology - Surgery - Orthopaedics - Intensive care - Accident and emergency - Other, namely: | |
| A6. | What is your position in this hospital? Give 1 answer which describes your position best. | - Nursing assistant/ auxiliary nurse - Trainee nurse - Qualified nurse - Physician’s Assistant / Nurse Practitioner - Physician - Physician in training for specialist (fellow) - Medical specialist - Pharmacist - Administrative employee / Secretary - Physiotherapist / Occupational therapist / Speech therapist - Medical analyst / laboratory technician - Radiotherapy technician - Function department employee (for example heart function, lung function, EEG) - Management - Other, namely: | |
| A7. | How long have you been working within your current specialism or this position? | - less than 3 months - 3 months to 1 year - 1 to 5 years - 6 to 10 years - 11 to 15 years - 16 to 20 years - 21 years or more | |
| A8. | How many hours per week do you usually work in this hospital? | - less than 20 hours per week - 20 to 39 hours per week - 40 to 59 hours per week - 60 or more hours per week | |
| A9. | Based on your position within this hospital, do you usually have direct interaction or contact with patients? | - YES, I usually DO have direct interaction or contact with patients - NO, I usually DO NOT have direct interaction or contact with patients | |
| Completion instructions Tripod Delta HC  Please carefully read the instructions below before starting to fill in the questionnaire.  Whilst deciding on your answer **ONLY** think about the situation at your department during the **LAST HALF YEAR** (anything that happened longer ago is no longer relevant to this research).  Please indicate for each statement the extent to which this statement applies to your department.  For this you can chose from the answer possibilities below:  *1 Strongly disagree*  *2 Disagree*  *3 Neutral*  *4 Agree*  *5 Strongly agree*  *NA Not applicable or no opinion*  We use the term 'healthcare staff' in this questionnaire. We mean all care providers and disciplines which are involved in direct patient care. | | | |
| 1. Less experienced healthcare staff is given work without clear working instructions. | | |  |
| 2. Tasks are inadequately carried out, or not carried out at all, because the knowledge and skills of the healthcare staff is insufficient. | | |  |
| 3. New healthcare staff is sufficiently monitored during their work. | | |  |
| 4. The executing staff have sufficient qualifications to carry out their work. | | |  |
| 5. Inexperienced healthcare staff is given sufficient time to gain practical experience. | | |  |
| 6. Superiors have the competence to carry out their work. | | |  |
| 7. The management provides sufficient opportunities to attend courses. | | |  |
| 8. There is an instruction programme for new healthcare staff which covers all important aspects of their work. | | |  |
| 9. My application for a course or training programme is often rejected without clear reasons. | | |  |
| 10. It happens often that I apply for a course or training programme, but cannot attend due to capacity problems at the department. | | |  |
| 11. Thanks to the training policy it is clear which training programmes I can attend and when. | | |  |
| 12. I sometimes have doubts about the professional competence of a colleague. | | |  |
| 13. There is always sufficiently experienced healthcare staff present at the department. | | |  |
| 14. I regularly have to redo the work of an inexperienced colleague because it was not carried out correctly. | | |  |
| 15. All important positions at my department are filled by qualified healthcare staff. | | |  |
| 16. I often received conflicting information from different sources without knowing which source is correct. | | |  |
| 17. When I am searching for specific information (for example a procedure) I often don't know where to find this. | | |  |
| 18. My questions are always answered within a reasonable period of time (from, for example, supporting departments or specialists). | | |  |
| 19. I often receive important information about changes in working methods and arrangements through the grapevine instead of through the official route. | | |  |
| 20. I have sometimes made a mistake because the necessary information had not been correctly conveyed. | | |  |
| 21. Important information is often sent to the wrong department in the hospital. | | |  |
| 22. I often receive so much information that I have to ignore (part of) this information. | | |  |
| 23. I am often confronted with a code or abbreviation the meaning of which I don't know and cannot easily find out. | | |  |
| 24. In my work area there is often so much noise that I cannot understand important information. | | |  |
| 25. Generally I can reach other departments easily by telephone. | | |  |
| 26. I sometimes do not receive a letter or email with important information because I was omitted from the mailing list by mistake. | | |  |
| 27. I often receive outdated information. | | |  |
| 28. When I ask something from different colleagues I get a different answer from each of them. | | |  |
| 29. I am aware of departments within my hospital that do not give me information when I ask for this, without good grounds. | | |  |
| 30. Patient information is often sent too late to a department. | | |  |
| 31. The tasks are not properly coordinated between departments so that work is carried out twice. | | |  |
| 32. Relatively unimportant matters take up too much of my time. | | |  |
| 33. I have sometimes been held wrongly responsible for something. | | |  |
| 34. I sometimes have to take decisions although I know that they are not part of my responsibilities. | | |  |
| 35. Management sometimes give me orders which in my opinion are unnecessary. | | |  |
| 36. My superior has sometimes reversed a decision I made without consulting with me. | | |  |
| 37. The management has persevered with an unusual policy. | | |  |
| 38. I often have to work outside my normal working hours due to poor cooperation. | | |  |
| 39. I have the sufficient competence to carry out my work properly. | | |  |
| 40. It has happened before that I did not know to whom I could delegate tasks. | | |  |
| 41. It has happened before that I disagreed with a specific approach but did not dare speak about this with my superior. | | |  |
| 42. It is always clear in my work situation who is responsible for what. | | |  |
| 43. There are sometimes complaints made against me although I was not responsible for what had happened. | | |  |
| 44. I sometimes have carried out the same work twice due to miscommunication between departments. | | |  |
| 45. There is sufficiently qualified healthcare staff present to carry our all necessary work in my department. | | |  |
| 46. I sometimes carry out work against my will, under pressure from my superior. | | |  |
| 47. I have to deal with unworkable procedures in my work. | | |  |
| 48. A discussion has arisen before about a decision to be taken as a result of unclear procedures. | | |  |
| 49. Necessary maintenance work has been postponed due to high costs. | | |  |
| 50. I always have a sufficient budget available to carry out my work properly. | | |  |
| 51. At busy times I have to carry out more work than my actual task requires of me. | | |  |
| 52. There are procedures in force that are too cumbersome to work with. | | |  |
| 53. I work together with people who have been employed on the basis of financial considerations and not on the basis of their qualities. | | |  |
| 54. Management has failed before to resolve a clear risk situation because it is economically cheaper (for example a defect of equipment, colleagues who do not want to communicate with each other etc.) | | |  |
| 55. I report all incidents in accordance with the usual procedure at my department. | | |  |
| 56. Problems have arisen regarding adherence to procedures that have been implemented too quickly. | | |  |
| 57. There is always sufficiently qualified healthcare staff at the department to carry out the work properly. | | |  |
| 58. I have had to sacrifice my lunch break before because there is a shortage of staff. | | |  |
| 59. Sometimes I dare not discuss a complaint with my superior because I am afraid of his/her reaction. | | |  |
| 60. Certain informal rules within the hospital are in conflict with formal rules. | | |  |
| *Explanation:* The following questions are about working procedures. Procedure means a description of a working method, working process, guideline or system. This research is in particular about working procedures, which describe how a task must be carried out by healthcare staff. For example, think of a working procedure about the double-checking of medication, calculation of doses, putting in a drip, standards of competence for the use of equipment, requests for laboratory tests etcetera. | | |  |
| 61. Because procedures are insufficiently clear, I sometimes have to act according to my own discretion. | | |  |
| 62. There are rules in the hospital which can be interpreted in different ways. | | |  |
| 63. I have sometimes been confronted with a procedure, the meaning of which was completely unclear to me. | | |  |
| 64. I sometimes have had extra work for a relatively simple task because I had to follow a cumbersome procedure. | | |  |
| 65. I have sometimes searched for information which I subsequently found spread over different places. | | |  |
| 66. I have sometimes been confronted with a procedure which was formulated in a such a complex way that I could not oversee what would happen if I would follow the procedure. | | |  |
| 67. The procedures which I need for my work link up with practice. | | |  |
| 68. I sometimes work with instruments or equipment for which a clear manual is lacking. | | |  |
| 69. I have sometimes ended up in a situation whereby it was unclear if a procedure existed about how to act in such a situation. | | |  |
| 70. I have sometimes been confronted with new procedures which were not workable. | | |  |
| 71. I have sometimes not been able to find a procedure which I needed at that time. | | |  |
| 72. I have sometimes had to use a procedure which was so unclear that I had to determine myself how I had to act. | | |  |
| 73. There have sometimes been procedures concerning my work which had been changed without me being informed about this. | | |  |
| 74. I sometimes have had to deal with procedures the authors of which clearly had no understanding of how it would function in practice. | | |  |
| 75. It has happened that I did not understand a procedure due to the unclear lay-out. | | |  |

**Thank you for filling in the questionnaire!**

If you have any comments about the questionnaire then you can note these down below.

Tripod Delta HC ©NIVEL and Stichting Tripod Foundation
